# Dietary *Fructus sophorae* extracts supplementation improved production performance, antioxidant capacity, and intestinal microbiota in broiler chickens

**DOI:** 10.3389/fvets.2025.1735065

**Published:** 2026-01-16

**Authors:** Xiyi Yang, Yan Zheng, Peihua Wei, Jiandong Wei, Xuejun Yuan, Shuzhen Jiang, Weiren Yang, Ning Jiao

**Affiliations:** 1 Key Laboratory of Efficient Utilization of Non-Grain Feed Resources (Co-construction by Ministry and Province), Ministry of Agriculture and Rural Affairs, College of Animal Science and Technology, Shandong Agricultural University, Tai’an, China; 2 Shandong Dezhou Shenniu Pharmaceutical Co., Ltd., Dezhou, China; 3 Qingdao Huanshan Biotechnology Co., Ltd., Qingdao, China; 4 College of Life Sciences, Shandong Agricultural University, Tai’an, China

**Keywords:** antioxidation, broiler, intestinal microbiota, performance, sophorae

## Abstract

**Introduction:**

This study was conducted to examine the effects of *Fructus sophorae* extracts (SE) on the production performance, serum biochemistry and antioxidant, intestinal morphology, and cecal microbiota of broilers.

**Methods:**

A total of 1,088 1-day-old Arbor Acres (AA) broiler chickens were randomly assigned to four treatment groups with 8 replicates each and 34 chickens per replicate. Broilers received basal diets supplemented with 0 (CON), 100 (SE100), 150 (SE150), and 200 (SE200) mg/kg SE for 42 days, respectively.

**Results and discussion:**

The results showed that SE had no significant effect on the growth performance of broilers. However, SE supplementation significantly increased the organic matter and crude ash metabolic rates but decreased serum alkaline phosphatase activity (*p* < 0.05). In addition, 150 and 200 mg/kg SE supplementation increased serum total protein and total cholesterol contents (*p* < 0.05). SE supplementation also improved the antioxidant capacity by decreasing serum and liver malondialdehyde contents and by increasing serum glutathione peroxidase and liver superoxide dismutase (SOD) activities (*p* < 0.05). On the other hand, 150 and 200 mg/kg SE supplementation increased serum SOD activity (*p* < 0.05). Moreover, SE supplementation improved liver morphology. In addition, 150 and 200 mg/kg SE supplementation improved duodenal and ileal morphology by increasing villus height and villus height to crypt depth ratio (*p* < 0.05). Furthermore, SE supplementation balanced the intestinal microbiota composition and improved the microbial diversity. In conclusion, dietary 150 mg/kg SE supplementation could improve nutrient utilization efficiency, biochemical metabolism, antioxidant capacity, and intestinal and liver health in broilers, considering feed cost. This study provides a basis for SE application in broiler production

## 1 Introduction

Broilers in a high-density farming environment are susceptible to oxidative stress-induced growth retardation and immune dysfunction (1). With the sustained growth in global poultry consumption, the broiler industry has raised demands for efficient and safe production methods. Traditional antibiotics as growth promoters have reduced application due to food safety concerns such as bacterial resistance and drug residues, making the development of natural plant extracts with green, non-toxic properties a key research focus (2). Studies have demonstrated that certain plant extracts can enhance broiler nutrient metabolism by activating digestive enzyme activity and improving intestinal absorption capacity (3). It is well-known that plant polyphenols can induce anti-oxidative and cytoprotective effects by inducing nuclear factor erythroid 2-related factor-2 (Nrf2) (4). Additionally, the potential beneficial effects of polyphenols on production performance and their anti-oxidative and anti-inflammatory properties could be mediated by their positive effect on the microbial composition and diversity in broilers (5). In particular, 35 mg/kg *piper aduncum* polyphenols and flavonoids improved gut health, immune and anti-inflammatory activity, and performance indices of broiler chickens (6). Moreover, dietary supplementation with 200, 400, and 600 mg/kg *terminalia chebula* extract, which is enriched in tannins, phenolic acids, flavonoids, and polysaccharides, enhanced anti-oxidant capacity and gut health by increasing microbiota richness and diversity (7).

The *Fructus sophorae* extracts (SE), medicinal parts of the legume family Sophora, contain active components such as flavonoids (rutin and quercetin), saponins, and polysaccharides. In the present study, SE primarily includes 15.32 g/kg each of matrine and oxymatrine and 13.78 g/kg of rutin. These compounds have long been used in traditional medicine for anti-inflammation, antioxidant, and metabolic regulation (8). Modern pharmacological research has found that locust flowers contain a lot of flavonoids and polysaccharides, including rutin, quercetin, and locust flower polysaccharide, which have anti-inflammatory, antioxidant, hemostatic, and immune enhancement effects (9). It has been reported that dietary 200 mg/kg rutin supplementation enhanced the anti-oxidative capacity in oxidized oil challenge broilers (10), and the anti-oxidative effects of rutin were achieved by inhibiting MAPK/NF-κB signaling (11). In addition, 200 mg/kg quercetin alleviated inflammation by modulating gut microflora in broilers (12). Dietary saponin-rich soapnut (*Sapindus mukorossi*) shell powder (100, 150, and 200 mg/kg) could improve the immunity and welfare of broilers without affecting the growth performance negatively (13). Therefore, the optimal dose and effects of SE for broiler health should be studied due to its widespread use as a feed additive.

According to a previous study, plant polyphenols dosage was varied at 10 ~ 600 mg/kg (6, 10, 13–15) according to the status of broilers. Therefore, considering the active component composition of SE, this study was conducted to explore the effects of dietary supplementation at 100, 150, and 200 mg/kg SE on growth performance, serum antioxidant levels, intestinal morphology, and microbial community structure under normal feeding conditions in broilers. This would provide a theoretical basis and practical guidance for developing novel green feed additives and promoting healthy broiler production.

## 2 Materials and methods

### 2.1 Ethics statement

The broilers were fed according to the care and use of laboratory animals as stipulated by the Animal Nutrition Research Institute of Shandong Agricultural University and the Ministry of Agriculture of China (SDAUA-2021-081).

### 2.2 *Fructus sophorae* extracts

The *Fructus sophorae* extracts (SE) were provided by Shandong Shenniu Pharmaceutical Co., Ltd. (Dezhou, China). The contents of matrine and oxymatrine each were ≥ 0.8%, and the rutin content was ≥ 8 mg/g. High-performance liquid chromatography (HPLC) was used to measure the contents of matrine, oxymatrine, and rutin in SE, which revealed 15.32 g/kg each of matrine and oxymatrine and 13.78 g/kg of rutin, respectively.

### 2.3 Experimental design, animals, and management

In total, 108 1-day-old Arbor Acres broilers (body weight = 43.19 ± 0.61 g) were randomly assigned to four treatments in a completely randomized design with 8 replicates (34 chickens per replicate). The treatments included basal diet (CON) and basal diet supplemented with 100 (SE100), 150 (SE150), and 200 (SE200) mg/kg SE, respectively. The basal diet (Table 1) was formulated based on two-phase feeding programs (0–21 d and 21–42 d) recommended by The National Research Council (NRC, 1994) (16). The broilers were raised in cages and vaccinated according to the normal immunization program. In addition, they were provided *ad libitum* access to feed and water. The room temperature was kept at 35 °C for the first week, which then decreased by 1 °C every 2 days until reaching 21 °C.

**Table 1:** Ingredients and nutrient levels of the basal diet (%, air dry basis).

Ingredients	1–21 d	22–42 d	Nutrients^#^	1–21 d	22–42 d
Corn	50.00	49.40	Metabolizable energy MJ/kg	12.34	13.60
Soybean meal, 46% CP	28.20	24.60	Crude protein	23.00	21.50
Wheat flour	8.00	8.00	Calcium	0.90	0.83
Corn gluten meal	2.00	2.00	Total phosphorus	0.58	0.53
Cottonseed meal	5.50	5.50	Lysine	1.46	1.32
CaHPO4	0.90	0.80	Methionine	0.60	0.53
Pulverized Limestone	1.60	1.50	Threonine	1.01	0.92
Soybean oil	1.80	6.20			
Premix*	2.00	2.00			
Total	100.00	100.00			

* Supplied per kg of diet: Vitamin A 4525 IU, Vitamin D3 975 IU, Vitamin E 13 IU, Vitamin K3 2.5 mg, Vitamin B1 1.3 mg, Vitamin B2 4.0 mg, Vitamin B12 0.01 mg, pantothenic acid 7.50 mg, niacin 17.50 mg, biotin 0.1 mg, folic acid 0.6 mg, Mn (as MnSO4·H2O) 30 mg, Fe (as FeSO4·H2O) 40 mg, Zn (as ZnSO4·H2O) 30 mg, Cu (as CuSO4·5H2O) 4.25 mg, I (as KIO3) 0.135 mg, Se (as Na2SeO3) 0.1 mg.

^#^ Metabolizable energy was the calculated value, and the other nutrient levels were the analyzed values.

### 2.4 Growth performance

The average daily feed intake (ADFI), average daily gain (ADG), and feed-to-gain ratio (F/G) were determined by recording the daily feed intake and body weight (BW) on days 1, 21, and 42 per replicate.

### 2.5 Sample collection

The experiment on the availability of nutrients was based on the total fecal collection method. All excreta were continuously collected on 28–30 days. Feathers and shredded dry skin in excreta were carefully removed, then weighed, pooled by replicate, sampled, and stored at −20 °C for analysis.

The blood samples (5.0 mL) were collected into non-heparinized tubes at the end of the experiment by puncturing the wing vein with sterilized needles. Following sample collection, the blood samples were incubated at 37 °C for 2 h and centrifuged at 1,500 × g for 15 min, and the resulting serum was stored in 1.5 mL tubes at −20 °C for use later on (17).

The birds were then euthanized by cervical dislocation after bleeding. Subsequently, the liver was taken out and put into a 1.5-mL tube at −20 °C. The liver was homogenized in a pipette with ice-cold physiological saline (0.9%; pH = 7.4) at a ratio of 1:9 for 3 min using a mechanical homogenizer (Q24RC, Xin Beixi Biotechnology Co., Ltd., Jinan, Shandong, China) under an ice-water bath environment. Following centrifugation at 3,000 × g for 10 min at 4 °C, the supernatants were stored at −20 °C to estimate the antioxidant status (18). All samples were maintained on ice during the preparation process.

### 2.6 Apparent nutrient metabolic rate

The basal diet and excreta were analyzed for crude protein (CP) using the Kjeldahl method (CP = nitrogen × 6.25). Moreover, the excreta samples were dried at 62 ± 2 °C, and then, the dried samples were ground with a mortar and pestle, and stored in sealed containers for further analysis in terms of dry matter (DM), ether extract (EE), and crude ash (CA) as AOAC (2012) (19). The DM was measured by drying it at 103 ± 2 °C for 48 h, the EE and CA were measured by ether extraction and ashing at 550 °C in a muffle furnace (SX2-4-10; Longkou electric furnace manufacturer, Yantai, China). The following formula was used to calculate the metabolic rate of nutrients:


Apparent metabolic rate=[(TNI−TNE)/TNI]×100%

TNI, the total nutrient intake (g) of DM, CP, CA, and EE; TNE, the total nutrients in excreta of DM, CP, CA, and EE.

### 2.7 Serum biochemical indexes

The COBUS MIRA Plus automatic biochemical analyzer (Roche Diagnostic System Inc., USA) was used to determine serum enzymes [aspartate aminotransferase (AST), alanine aminotransferase (ALT), alkaline phosphatase (ALP), and lactate dehydrogenase (LDH)] and serum metabolites [total protein (TP), albumin (ALB), urea nitrogen (SUN), glucose (GLU), triglyceride (TG), and total cholesterol (TC)].

### 2.8 Antioxidant enzymes in serum and liver

The content of malondialdehyde (MDA) and the activities of superoxide dismutase (SOD) and glutathione peroxidase (GSH-Px) in the serum and liver were measured with the kits manufactured by Nanjing Jiancheng Bioengineering Institute. The test was performed strictly following the instructions and the previous study (20). The microplate reader (multifunctional enzyme-linked immunosorbent assay reader Synergy 4, BioTek Inc., USA) was used to measure the optical density (OD) value, and the results were calculated according to the manufacturer’s provided formula.

### 2.9 Histological structure of liver and intestine

The analysis of morphology of the liver and intestine was analyzed according to the previous study (21). Briefly, at the end of the experiment, samples of liver and intestine were fixed in a 4% paraformaldehyde solution for 24 h and then trimmed to a thickness of about 1 cm. The tissues were placed into embedded cassettes and rinsed with water to get rid of the paraformaldehyde solution. Then, the tissue blocks were dehydrated with 70, 80, 85, 90, 95, and 100% ethyl alcohol for 2 h. Afterward, the blocks were embedded in liquid paraffin and left for 12 h. After embedding, the blocks were cut into 6 μm sections by a microtome. The sections were put on the slides and dried at 37 °C. The sections were stained with hematoxylin and eosin (H&E), dripped with neutral gum, covered with cover glass, and put into 37 °C oven for drying. A microscope photography system (Nikon Eclipse 80i) was used to examine the sections, and the pictures were obtained by means of a DP25 digital camera. The villus height (VH) and crypt depth (CD) were measured from eight pictures per group and 40 villi and crypts per picture. Finally, the ratio of VH to CD (VH/CD) was calculated.

### 2.10 Microbial analysis

The microbial analysis was based on the earlier research (22). The CTAB method was used to extract the genomic DNA, and agarose gel electrophoresis was used to determine the concentration and purity. A suitable amount of the sample was suctioned into a sterile, enzyme-free tube and then diluted to 1 ng/μL using sterile water. PCR amplification was performed using specific primers containing a Barcode and high-efficiency and high-fidelity enzymes to make sure that the amplification is efficient and accurate using the final diluted sample DNA as a template. The sequencing area was the V3-V4 region, and the primer sequences used were 341F (CCTAYGGGRBGCASCAG) and 806R (GGACTACNNGGGTATCTAAT). The PCR products were detected by agarose gel electrophoresis at a 2% concentration. The qualified products were purified using magnetic beads. Following equalizing the concentrations of the PCR products through microplate reader-based quantification, electrophoresis detection was performed. The Qiagen gel extraction kit (QIAGEN, Hilden, Germany) was employed to recover the products in the target bands. The TruSeq® DNA PCR-Free Sample 3 Preparation kit (Illumina, San Diego, CA, USA) was used to construct the library, and it was quantified using Qubit (Thermo Fisher Scientific, Waltham, MA, USA) and Q-PCR (Thermo Fisher Scientific, Waltham, MA, USA). Once it met the qualification criteria, sequencing was carried out on the instrument using the NovaSeq6000. The original data underwent rigorous screening and filtering. Then, all samples were classified into Operational Taxonomic Units (OTUs) with a similarity threshold 97%. These OTUs were then clustered and species classified.

### 2.11 Statistical analysis

The experimental data were analyzed using SAS 9.4 software to obtain the statistical analysis of the data, and s one-way ANOVA was used to compare the differences between treatments. In addition, the General Linear Model (GLM) was used for linear and quadratic regression analyses of the experimental data. Tukey’s method was used for the measured data’s multiple comparisons with a significance level of a *p*-value of < 0.05. GraphPad Prism (version 9, La Jolla, CA, United States) was used to draw figures.

## 3 Results

### 3.1 Growth performance

The impact of SE on broiler growth performance is shown in Table 2. There were no significant effects of SE (100, 150, and 200 mg/kg) on ADFI, ADG, and F/G in broilers (*p* > 0.05).

**Table 2:** The effects of *Fructus sophorae* extracts on the growth performance of broiler chickens.

Items	Control	SE100	SE150	SE200	SEM	*p*-value		
						Treatment	Linear	Quadratic
1–21 d								
ADFI, g	50.95	50.99	50.12	50.42	0.205	0.386	0.190	0.408
ADG, g	40.62	40.69	40.05	40.82	0.169	0.403	0.988	0.599
F/G	1.25	1.25	1.25	1.24	0.004	0.384	0.135	0.225
22–42 d								
ADFI, g	138.21	134.90	135.87	134.62	0.745	0.358	0.161	0.304
ADG, g	88.09	86.95	87.76	87.23	0.557	0.889	0.704	0.894
F/G	1.57	1.55	1.55	1.54	0.005	0.423	0.094	0.213
1–42 d								
ADFI, g	94.60	92.92	92.37	93.15	0.411	0.307	0.205	0.160
ADG, g	64.39	63.79	63.64	64.29	0.317	0.792	0.868	0.594
F/G	1.47	1.48	1.45	1.45	0.004	0.223	0.044	0.108

Control, SE100, SE150, and SE200 represent the basal diet supplemented with 0, 100, 150, and 200 mg/kg *Fructus sophorae* extracts, respectively. SE, *Fructus sophorae* extracts; ADFI, average daily feed intake; ADG, average daily gain; F/G, feed-to-gain ratio. SEM, total standard error of the means.

### 3.2 Apparent nutrient metabolic rate

The results of SE on apparent nutrient metabolic rate in broilers are presented in Table 3. As the SE (100, 150, and 200 mg/kg) concentration increased, the apparent metabolism of OM exhibited linear and quadratic reduction, while that of CA was linear and quadratic increase (*p* < 0.05). The apparent metabolism of OM and CA in SE100, SE150, and SE200 was much higher than that of the control (*p* < 0.05).

**Table 3:** The effects of *Fructus sophorae* extracts on the nutrient metabolism rate of broiler chickens (%).

Items	Control	SE100	SE150	SE200	SEM	*p-*value		
						Treatment	Linear	Quadratic
DM	85.23	85.21	85.12	85.20	0.159	0.997	0.906	0.986
OM	87.59^b^	88.96^a^	88.49^a^	88.92^a^	0.175	< 0.001	0.015	0.013
CP	80.13	80.96	80.88	80.90	0.247	0.625	0.326	0.449
EE	85.37	85.13	85.04	85.01	0.235	0.956	0.593	0.849
CA	47.15^b^	48.09^a^	48.33^a^	48.33^a^	0.145	0.003	0.002	0.001

Control, SE100, SE150, and SE200 represent the basal diet supplemented with 0, 100, 150, and 200 mg/kg *Fructus sophorae* extracts, respectively. SE, *Fructus sophorae* extracts; DM, dry matter; OM, organic matter; CP, crude protein; EE, ether extract; CA, crude ash. SEM, total standard error of the means. ^a,b^ Mean differ significantly (*p* < 0.05).

### 3.3 Serum biochemistry

The effects of SE on serum biochemistry in broilers are indicated in Table 4. With the increase in SE (100, 150, and 200 mg/kg) concentration, ALP activity increased linearly and quadratically (*p* < 0.05). The ALP activities in SE groups were much lower than those in the control (*p* < 0.05). Nevertheless, no significant differences in activities of ALT, AST, and LDH were found among all treatments (*p* > 0.05). In addition, with the increasing SE (100, 150, and 200 mg/kg) concentration, TP and TC levels in the serum of broilers were increased linearly and quadratically (*p* < 0.05; Table 5). In the SE150 and SE200 groups, TP and TC levels were significantly higher than in the control and SE100 groups (*p* < 0.05).

**Table 4:** The effects of *Fructus sophorae* extracts on serum enzymes in broiler chickens (U/L).

Items	Control	SE100	SE150	SE200	SEM	*p-*value		
						Treatment	Linear	Quadratic
ALT	4.76	4.75	4.76	4.74	0.077	0.999	0.939	0.997
AST	450.9	447.1	447.1	446.6	3.46	0.973	0.682	0.899
ALP	3123.8^a^	2932.2^b^	2961.3^b^	2856.6^b^	30.05	0.006	0.002	0.006
LDH	1675.0	1675.6	1674.5	1673.0	10.02	0.999	0.941	0.996

Control, SE100, SE150, and SE200 represent the basal diet supplemented with 0, 100, 150, and 200 mg/kg *Fructus sophorae* extracts, respectively. SE, *Fructus sophorae* extracts; ALT, alanine aminotransferase; AST, aspartate aminotransferase; ALP, alkaline phosphatase; LDH, lactate dehydrogenase. SEM, total standard error of the means. ^a,b^ Mean differ significantly (*p* < 0.05).

**Table 5:** The effects of *Fructus sophorae* extracts on serum metabolites in broiler chickens.

Items	Control	SE100	SE150	SE200	SEM	*p-*value		
						Treatment	Linear	Quadratic
TP, mol/L	40.91^b^	41.72^b^	43.95^a^	44.84^a^	0.412	< 0.001	< 0.001	< 0.001
ALB, mmol/L	7.37	7.31	7.29	7.19	0.047	0.615	0.187	0.418
SUN, mmol/L	0.76	0.76	0.76	0.78	0.003	0.302	0.074	0.168
GLU, μmol/mL	14.40	14.40	14.51	14.54	0.035	0.415	0.107	0.276
TG, mmol/L	1.15	1.13	1.13	1.13	0.011	0.894	0.585	0.763
TC, mmol/L	3.24^b^	3.30^b^	3.53^a^	3.59^a^	0.037	< 0.001	< 0.001	< 0.001

Control, SE100, SE150, and SE200 represent the basal diet supplemented with 0, 100, 150, and 200 mg/kg *Fructus sophorae* extracts, respectively. SE, *Fructus sophorae* extracts; TP, total protein; ALB, albumin; SUN, urea nitrogen; GLU, glucose; TG, triglyceride; TC, total cholesterol. SEM, total standard error of the means. ^a,b^ Mean differ significantly (*p* < 0.05).

### 3.4 Antioxidant parameter

Table 6 indicates the effects of SE on the antioxidant activity in the serum and liver. With the increase in SE (100, 150, and 200 mg/kg) concentration, the serum and liver levels of MDA showed a linear and quadratic decrease (*p* < 0.05). Concurrently, SOD activities in serum and liver, and GSH-Px activities in serum increased linearly and quadratically (*p* < 0.05). As for the serum antioxidant indexes, the MDA levels in the SE150 and SE200 groups were lower than those in the SE100 group (*p* < 0.05), and the MDA level in the SE100 group was lower than that in the control group (*p* < 0.05). Nevertheless, the activities of GSH-Px in the SE groups were higher than that in the control group (*p* < 0.05). Meanwhile, the activities of SOD in SE150 and SE200 were significantly higher than those in the control and SE100 groups (*p* < 0.05). Regarding liver antioxidant indexes, the MDA levels in the SE groups were lower than those in the control group (*p* < 0.05), whereas the activity of SOD exhibited an opposite trend.

**Table 6:** The effect of *Fructus sophorae* extract on the antioxidant activity of serum and liver in broiler chickens.

Items	Control	SE100	SE150	SE200	SEM	*p-*value		
						Treatment	Linear	Quadratic
Serum								
MDA, nmol/mL	4.61^a^	4.20^b^	3.94^c^	3.96^c^	0.074	< 0.001	< 0.001	< 0.001
SOD, U/mL	125.58^b^	129.97^b^	138.34^a^	138.54^a^	1.584	< 0.001	< 0.001	< 0.001
GSH-Px, U/mL	879.90^b^	930.79^a^	937.33^a^	961.07^a^	8.824	< 0.001	< 0.001	< 0.001
Liver								
MDA, nmol/mg prot	2.73^a^	2.56^b^	2.55^b^	2.58^b^	0.024	0.013	0.033	0.005
SOD, U/mg prot	43.68^b^	46.99^a^	48.52^a^	47.32^a^	0.513	< 0.001	0.003	< 0.001
GSH-Px, U/mg prot	28.42	28.53	28.58	28.72	0.093	0.751	0.262	0.545

Control, SE100, SE150, and SE200 represent the basal diet supplemented with 0, 100, 150, and 200 mg/kg *Fructus sophorae* extracts, respectively. SE, *Fructus sophorae* extracts; MDA, malondialdehyde; SOD, superoxide dismutase; GSH-Px, Glutathione peroxidase. SEM, total standard error of the means. ^a,b,c^ Mean differ significantly (*p* < 0.05).

### 3.5 Liver morphology

**Figure 1:**
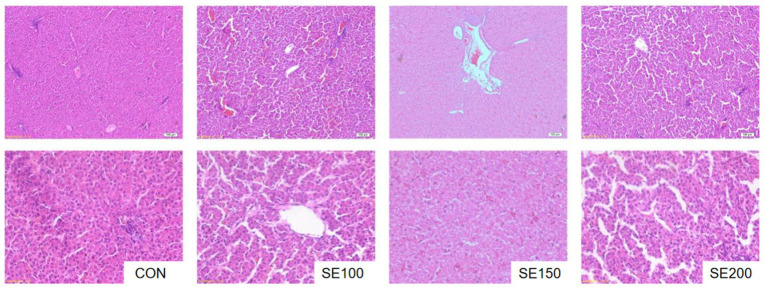
The effects of *Fructus sophorae* extracts on the morphological structure of the liver in broiler chickens. Control, SE100, SE150, and SE200 represent the basal diet with additions of 0, 100, 150, and 200 mg/kg SE, respectively.

The effects of SE on the liver morphology in broilers are demonstrated in Figure 1. In the control group, some hepatocytes were pushed to one side, accompanied by an increased presence of inflammatory infiltrates. The SE100 group had red blood cells in hepatic sinuses and had only a small infiltration of inflammatory cells, indicating that SE has the potential to alleviate liver injury and maintain the normal morphology of the liver tissue. The hepatocytes of the SE150 and SE200 groups exhibited uniform size, complete morphology, and neat arrangement, with uniform cytoplasm and clear nuclei. Both the liver sinuses and the liver cord structure were clearly visible. There was minimal bleeding, and no evident inflammatory infiltration was observed. Furthermore, the enlargement of the gap between liver sinusoids in SE200 suggested that SE improved the liver tissue structure of broilers.

### 3.6 Intestine morphology

**Figure 2:**
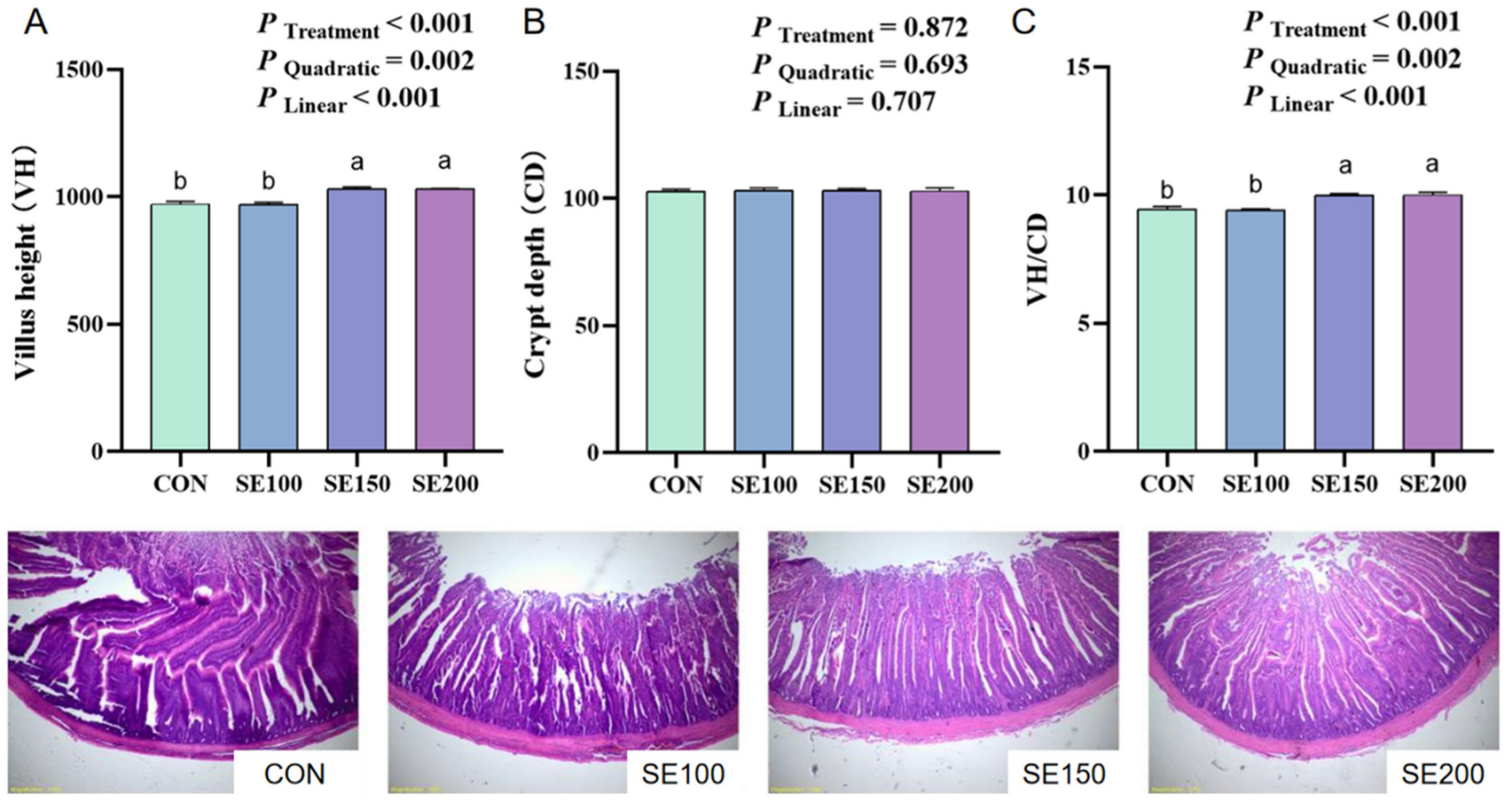
The effects of *Fructus sophorae* extracts on the duodenal morphology in broiler chickens. Control, SE100, SE150, and SE200 represent the basal diet with additions of 0, 100, 150, and 200 mg/kg SE, respectively. **(A)** Villus height (VH). **(B)** Crypt depth (CD). **(C)** VH/CD. ^a,b^ Mean differ significantly (*p* < 0.05).

**Figure 3:**
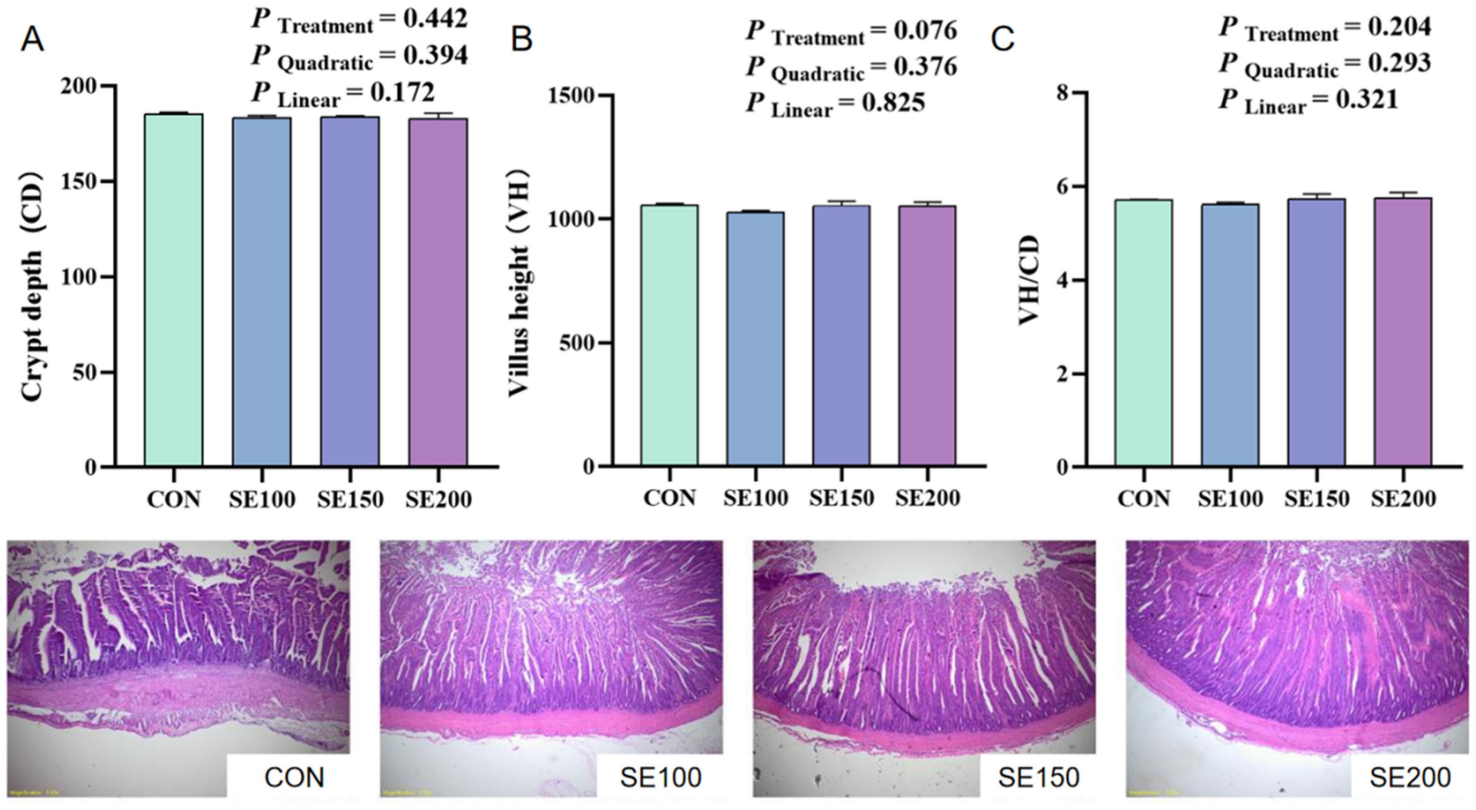
The effects of *Fructus sophorae* extracts on the jejunal morphology in broiler chickens. Control, SE100, SE150, and SE200 represent the basal diet with additions of 0, 100, 150, and 200 mg/kg SE, respectively. **(A)** Villus height (VH). **(B)** Crypt depth (CD). **(C)** VH/CD.

**Figure 4:**
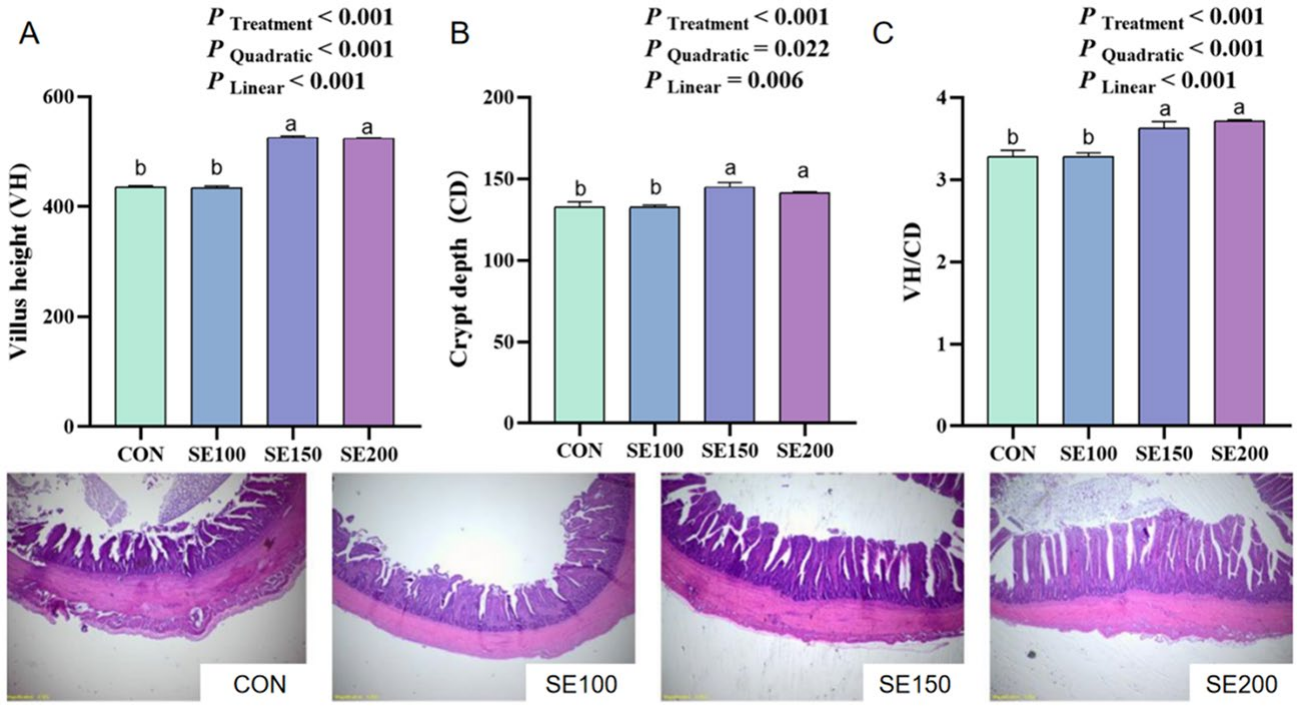
The effects of *Fructus sophorae* extracts on the ileal morphology in broiler chickens. Control, SE100, SE150, and SE200 represent the basal diet with additions of 0, 100, 150, and 200 mg/kg SE, respectively. **(A)** Villus height (VH). **(B)** Crypt depth (CD). **(C)** VH/CD. ^a,b^ Mean differ significantly (*p* < 0.05).

With the increasing SE (100, 150, and 200 mg/kg) supplementation, the VH and VH/CD of the duodenum increased linearly and quadratically (*p* < 0.05), and the VH and VH/CD in the SE150 and SE200 groups of the duodenum were much higher than those in the control and SE100 groups (*p* < 0.05; Figure 2). However, no significant effects of SE on the VH, CD, and VH/CD of the jejunum were found (*p* > 0.05; Figure 3). The VH, CD, and VH/CD of the ileum showed a linear and quadratic increase (*p* < 0.05). Moreover, VH, CD, and VH/CD in the SE150 and SE200 groups were higher than those in the control and SE100 groups (*p* < 0.05; Figure 4).

### 3.7 Cecal bacteria community

#### 3.7.1 Cecal microbial diversity

**Figure 5:**
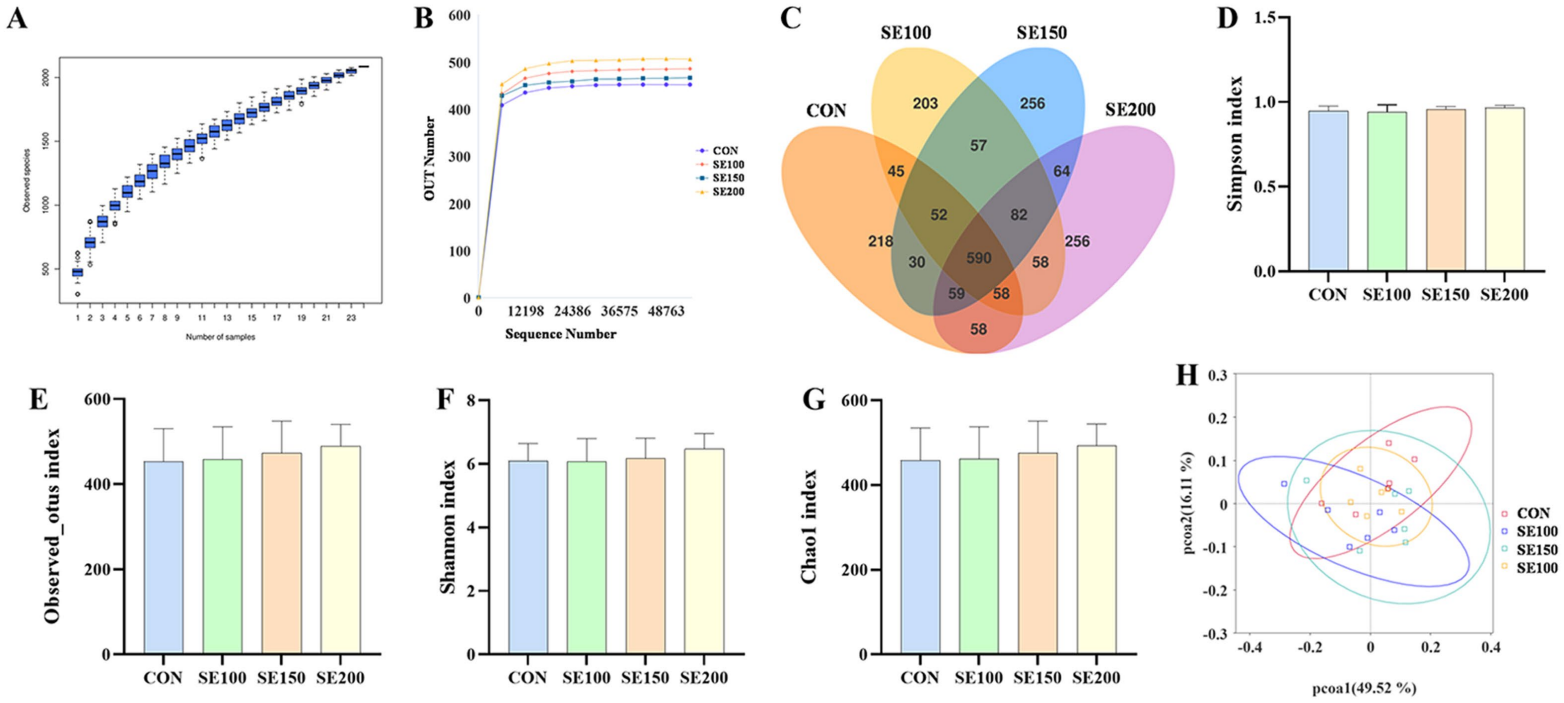
The effects of *Fructus sophorae* extracts on cecal microbiota diversity in broiler chickens. **(A)** Species accumulation boxplot of OTUs. **(B)** Dilution curve. **(C)** Venn diagram of OTUs. **(D)** Simpson index. **(E)** Observed_otus index. **(F)** Shannon index. **(G)** Chao1 index. **(H)** Beta diversity of cecal microbiota.

The species accumulation box plot of OTUs in the cecal microbiota (Figure 5A) can be used to predict the richness of species. The result presented that the trend of the box plot tended to be gentle, suggesting that the samples could fully reflect the richness of the community. Additionally, the sparsity curve approached the asymptote, which means that the sequencing amount of the sample was moderate and could accurately reflect the information of most of the microorganisms in the cecal content (Figure 5B). There are a total of 590 OTUs among all groups. Specifically, the control, SE100, SE150, and SE200 groups have 218, 203, 256, and 256 unique OTUs, respectively. The control and SE200, along with the SE100 and SE200 groups, had the highest number of OTUs, with 58 OTUs each. On the other hand, the control and SE150 groups had the lowest number of common OTUs, only 30 OTUs (Figure 5C).

The alpha diversity reflected intestinal flora richness and homogeneity. No significant differences in Observed_otus, Chao1, Shannon, and Simpson indexes were observed (*p* > 0.05; Figures 5D–G). Additionally, the outcome of the principal component analysis revealed that cecal microbiota composition of the four treatments was essentially similar, indicating that there was no significant variation in the microbial composition between groups (Figure 5H).

#### 3.7.2 Cecal microbial abundance

**Figure 6:**
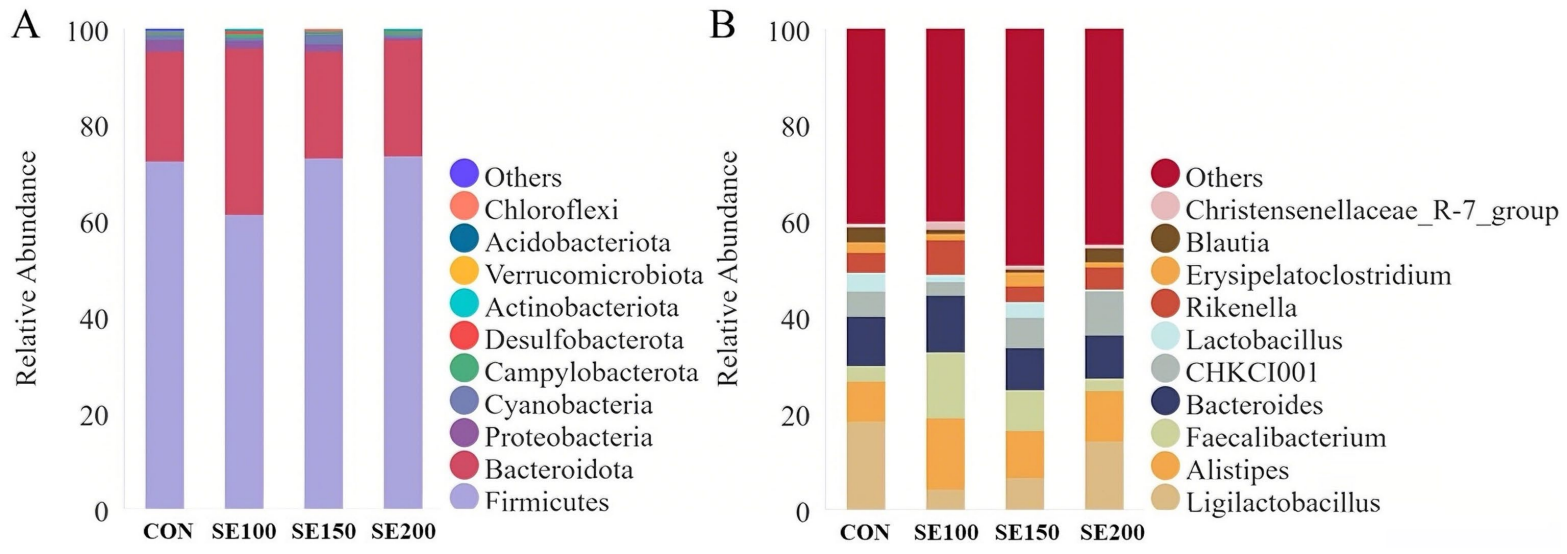
The effects of *Fructus sophorae* extracts on cecal microbiota abundance in broiler chickens. **(A)** The abundance of the TOP10 species at the phylum level. **(B)** The abundance of the TOP10 species at the genus level.

The abundance of cecal microbiota was reflected at the phylum and genus levels. Firmicutes had the highest relative abundance as a species, followed by Bacteroidota, and then Proteobacteria (Figure 6A). *Ligulactobacillus*, *Alistipes*, *Faecalibacterium,* and *Bacteroides* were the most abundant species in terms of their relative abundance at the genus level (Figure 6B). Following the comparison of the top 10 microbial abundance phyla and genera, there were no differences between treatments at both the genus and phylum levels of microbial communities.

#### 3.7.3 LEfSe analysis plot of cecal microbiota

**Figure 7:**
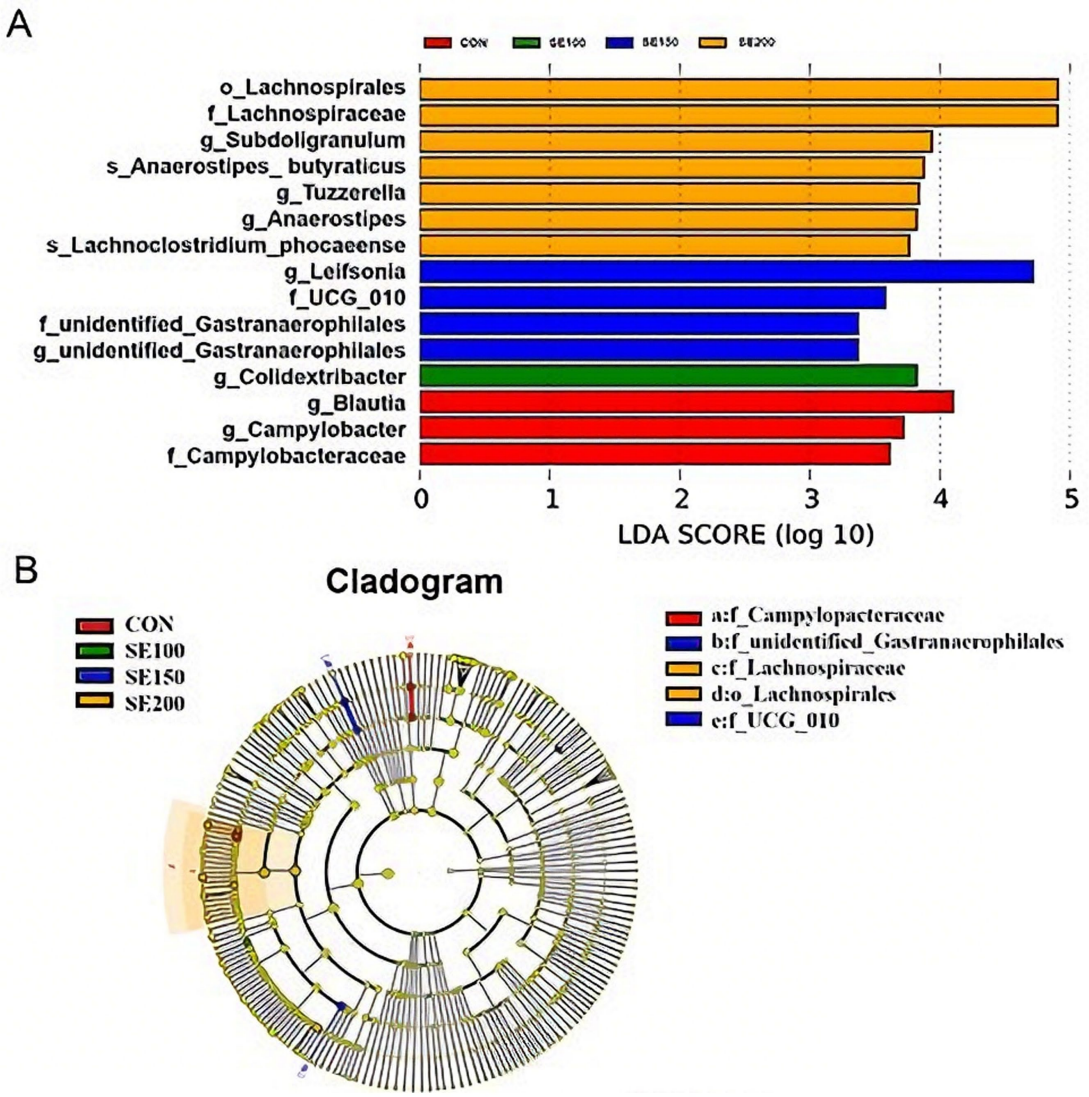
LEfSe analysis of cecal microbiota. **(A)** Histogram of LDA value distribution. **(B)** Evolutionary branching graph.

LEfSe analysis revealed that a total of 15 species demonstrated significantly different abundances. Compared with the control group, as indicated in Figure 7A, the abundances of *Lachnospirales*, *Lachnospiraceae*, *Subdoligranulum*, *Anaerostipes_butyraticus*, *Tuzzerella, Anaerostipes,* and *Lachnoclostridium_phocaeense* in the SE200 group were much higher. The abundances of *Leifsonia*, *UCG_010,* and *unidentified_ Gastroanaerobhilales* in the SE150 group were also much higher than the control group. In addition, the abundances of *Colidextribacter* in the SE100 group were much higher than in the control group. As shown in Figure 7B, the *Campylobacteraceae*, and *unidentified_ Gastroanaerobhilales* and *UCG_010* played a vital role in the control and SE150 groups, respectively, while the *Lachnospiraceae* and *Lachnospirales* played a vital role in the SE200 group.

## 4 Discussion

Current research findings concerning SE on the growth performance exhibit a general consensus. The majority of studies suggest that SE exerted a beneficial influence on animal growth. For instance, a study showed that the supplementation of quercetin (0.2, 0.4, or 0.6 g/kg) extracted from *Sophora japonica* flower in the diet increased ADG in broiler chickens (23). Similarly, another study demonstrated that dietary supplementation of sophora extract 10, 20, or 30 mg/kg increased spleen and bursa of fabricius weights, improved body weight gain, while reducing feed consumption in Korean Native Chickens (15). In addition, 1 mL of *sophora flavescens* aqueous extract (100 mg/mL) daily to broilers normalized weight gain by day 6 after *Eimeria tenella* infection (24). Nonetheless, the present study revealed that 100, 150, and 200 mg/kg SE supplementation exerted no notable influence on broiler growth performance, which was caused by different concentrations of SE supplementation or chicken breed.

The dietary nutrient apparent metabolic rate in broilers serves as a crucial indicator for evaluating their capacity to digest and absorb nutrients effectively, which indicates growth performance and overall health of broilers. Study reported quercetin extracted from *Sophora japonica* flower supplementation (0.2, 0.4, or 0.6 g/kg) improved linearly apparent DM digestibility and apparent energy retention (23). In addition, dietary 0.6 g/kg flavonoid blend supplementation linearly and quadratically increased crude protein metabolism compared to the control group in broiler chickens (25). The results of this experiment revealed that SE (100, 150, and 200 mg/kg) supplementation significantly enhanced the apparent metabolism of organic matter and crude ash in broilers, aligning with previous research reports. This can be ascribed to the flavonoids present in SE, which facilitate the growth of intestinal villi in broilers, augment the contact surface area with chyme, and promote the secretion of digestive enzymes, thereby enhancing the apparent metabolism of nutrients.

The broiler health was also reflected by serum chemistry. The serum activity of ALP was commonly observed in the broiler growth and development period, as well as in cases of abnormal liver function. Studies showed that dietary supplementation with 350 mg/kg flavonoid luteolin for 4 weeks significantly decreased the ALP level in broiler chickens (26). In addition, it reported that dietary supplementation with 400, 800, and 1,600 mg/kg bamboo leaf flavonoid reduced serum ALP in cyclic heat stress broilers (27). Similarly, a notable finding in the present study is the decrease in serum ALP activity following the administration of 100 ~ 200 mg/kg SE. Compared with previous studies, the low dosage used may be due to broilers being in a normal environmental state rather than a stress state in the current study. This effect is likely because of the bioactive components in the extract, such as flavonoids, which have the potential to regulate metabolic processes or exert protective effects on the hepatobiliary system.

As the final product of lipid peroxidation, MDA can be a direct indicator of oxidative damage in the organism (10). Meanwhile, SOD can scavenge superoxide anion radicals, and GSH-Px has the potential to reduce the build-up of hydrogen peroxide and lipid peroxides (28). Study demonstrated that supplementation with 450 mg/kg kudzu-leaf flavonoids promoted the anti-oxidant capacity by increasing SOD activity and decreasing MDA content (14). Study found that astragalus polysaccharide plant extract could significantly enhance serum GSH-Px activity and decrease serum MDA content in hens (29). These findings align with the present study, in which SE supplementation resulted in a drastic decline in MDA concentration in serum and liver tissues. Concurrently, SOD activities in serum and liver, as well as GSH-Px activities in serum, were elevated. Similarly, a study reported that 300 mg/kg bw/day phenolics isolated from Sophora interrupta Bedd could restore the activities of SOD and GSH-Px in NDV-induced oxidative stress in chickens (30). This thus indicated that SE could improve antioxidant capacity, thereby mitigating the cumulative damage caused by reactive oxygen species. Studies showed the mechanism of plant bioactive components’ anti-oxidative effects mediated through the activation of the Nrf2 signaling pathway or inhibiting MAPK/NF-κB signaling (4, 11). Therefore, further study should be conducted to clarify the mechanism of SE in regulating antioxidants in broilers.

The effective execution of normal hepatic function relies on the structural integrity of hepatic tissue. It has been reported that, as a component of SE, dietary 500 mg/kg of rutin could ameliorate liver necroptosis through suppressing oxidative stress and the MAPK/NF-κB pathway in chickens (11). Moreover, 125 mg/kg flavonoids of *rhizoma jeffersoniae* (97% rutin) protect chicken liver against toxicity through the interaction of the microbiota–gut–liver axis mechanisms (31). Study found that sophoricoside at 80 and 160 mg/kg·BW was observed to reduce body weight and liver weight, hepatic cholesterol, triglyceride levels, and MDA in HF-fed mice and alleviated liver morphology injury (32). In the present study, SE contains flavonoids (rutin and quercetin), saponins, and polysaccharides. The present study observed that 100 mg/kg SE improved the morphology of hepatic tissue in broilers, a finding consistent with the well-documented hepatoprotective properties of flavonoids. Moreover, 150 and 200 mg/kg SE reduced inflammatory infiltration and restored the normal hepatocyte morphology, which accompanied enhanced liver anti-oxidative capacity. These findings were consistent with the previous finding, which showed that bush sophora root polysaccharides upregulated SOD2 expression, thereby protecting against hepatotoxicity (33). Similarly, a study reported that intraperitoneal injection of 30 mg/kg sophoricoside alleviated hepatic inflammation and maintained hepatocyte integrity by inhibiting NF-κB pathways (34). The dose of SE used in the study for hepatoprotection was also according to the previous studies (31, 32, 34) and the active component composition in SE. Additional research was required to verify its efficacy and the molecular mechanism of SE in terms of hepatoprotective characteristics under stress conditions.

Furthermore, the morphology and microbial composition, acting as critical determinants of intestinal health, were determined in this study. Intestinal health is important for nutrient absorption, enhancing anti-oxidative capacity, and animal health (35). The VH and CD were morphological measures that are typically employed to correctly measure the wellbeing and functionality of chickens (36, 37). We found that the ileal VH, CD, and VH/CD displayed a gradual elevation with the increase of SE levels. Similarly, a previous study confirmed that an intraperitoneal injection of 0.2 mL sophoricoside (60 mg/kg) could ameliorate colitis and protect intestinal barrier function by reducing enterocyte apoptosis (38). Additionally, it was observed that 100 mg/kg xymatrine from *Sophora flavescens* significantly enhanced colonic VH and VH/CD (39). The beneficial effects of plant bioactive compounds in enhancing gut morphology are likely through mechanisms involved in promoting short-chain fatty acid (SCFA) synthesis and epithelial cell renewal (40). Therefore, further studies should validate whether these morphological adaptations could translate to nutrient absorption or barrier function improvement and explore the mechanism of SE in intestinal function.

Moreover, microbiota in the cecum form a dynamically balanced community, which has important functions in the metabolism of nutrients of the host, immune regulation, and intestinal barrier maintenance (3). When the cecum flora is balanced and functioning properly, broilers could use the nutrients more efficiently (41). Firmicutes ferment dietary fiber to produce SCFAs that can interact with the mucosa of the intestine and possibly contribute to the homeostasis of the host (42). Meanwhile, Bacteroidota can degrade complex carbohydrates and are resistant to pathogen invasion (43). Firmicutes and Bacteroidota predominantly dominated all groups in the present study, which suggested that SE could improve gut microbiota composition. The increase in the Firmicutes to Bacteroidota ratio indicates enhanced animal physiological function (3). Similarly, in the present study, the relative abundance of Firmicutes was higher than that of Bacteroidota in the SE-treated groups, indicating that SE could enhance broiler health. Future studies should aim to extend the duration of interventions or incorporate microbial metabolic profiling to elucidate the interaction mechanisms between SE and broiler gut microbiota. The interventions, such as host diet, age, and antibiotics, could influence the structural homeostasis of the host microbial community (44). Nevertheless, we found no significant difference between the SE-treated and control groups in terms of microbiotal richness and *α* diversity. At the same time, the study reported that dietary supplementation with 500 mg/kg herbal mixtures, including ginseng and artichoke, did not have a significant effect on the microbial richness and diversity of chickens (45). This may be because the concentration of SE used in this study cannot induce changes in microbiota. Instead, it may have induced more subtle, specific compositional changes that are not reflected in alpha diversity metrics. *Lachnospiraceae* was known to produce butyrate, which could provide energy and maintain intestinal health and functions (46). LEfSe analysis of our study revealed that 200 mg/kg SE significantly enriched *Lachnospiraceae*. These results indicated that SE could improve intestinal health by modulating gut morphology and microbiota involved in enhancing SCFAs production.

## 5 Conclusion

Dietary supplementation of 100, 150, and 200 mg/kg SE improved the OM and CA utilization as well as antioxidant capacity by reducing serum and liver MDA content as well as increasing SOD and GSH-Px activities. In addition, SE maintained liver health by decreasing serum ALP content and improving liver morphology. Moreover, the duodenal and ileal morphology was improved, and the cecal microbiota community involved in enhancing SCFAs production was balanced upon SE supplementation. In summary, SE regulated the microbiota–gut–liver axis in broilers, which provides an important strategy for promoting broiler production. Considering the effectiveness and feed cost, 150 mg/kg is the optimal dose for SE supplementation in the broiler diet. Further study should focus on a certain signaling pathway and molecule of SE in regulating the microbiota–gut–liver axis, thereby providing a theoretical framework for its application and providing clarification on its mechanism of action as a feed additive in broiler production.

## Data Availability

The datasets presented in this study can be found in online repositories. The data presented in the study are deposited in the http://www.ncbi.nlm.nih.gov repository, accession number PRJNA1357099.
