# Correction: Dietary *Fructus sophorae* extracts supplementation improved production performance, antioxidant capacity, and intestinal microbiota in broiler chickens

**DOI:** 10.3389/fvets.2026.1824404

**Published:** 2026-05-15

**Authors:** Xiyi Yang, Yan Zheng, Peihua Wei, Jiandong Wei, Xuejun Yuan, Shuzhen Jiang, Weiren Yang, Ning Jiao

**Affiliations:** 1 Key Laboratory of Efficient Utilization of Non-Grain Feed Resources (Co-construction by Ministry and Province), Ministry of Agriculture and Rural Affairs, College of Animal Science and Technology, Shandong Agricultural University, Tai'an, China; 2 Shandong Dezhou Shenniu Pharmaceutical Co., Ltd., Dezhou, China; 3 Qingdao Huanshan Biotechnology Co., Ltd., Qingdao, China; 4 College of Life Sciences, Shandong Agricultural University, Tai'an, China

**Keywords:** antioxidation, broiler, intestinal microbiota, performance, *sophorae*

There was a mistake in Figure 6 and Table 6 as published.

Figure 6 inadvertently contains a watermark. During the final figure assembly using graphics editing software, a translucent software identification mark (often referred to as a watermark) was inadvertently preserved on the image due to an oversight in the layer merging or export settings. We want to emphasize that this artifact is solely a remnant from the image processing software and is unrelated to, nor does it indicate, the use of any AI-based content generation tools for data acquisition or analysis. The underlying raw data and the scientific conclusions of the figure remain entirely unchanged and valid. The presence of this watermark was a technical negligence in the final graphic preparation stage.

The corrected Figure 6 appears below:

**Figure 6:**
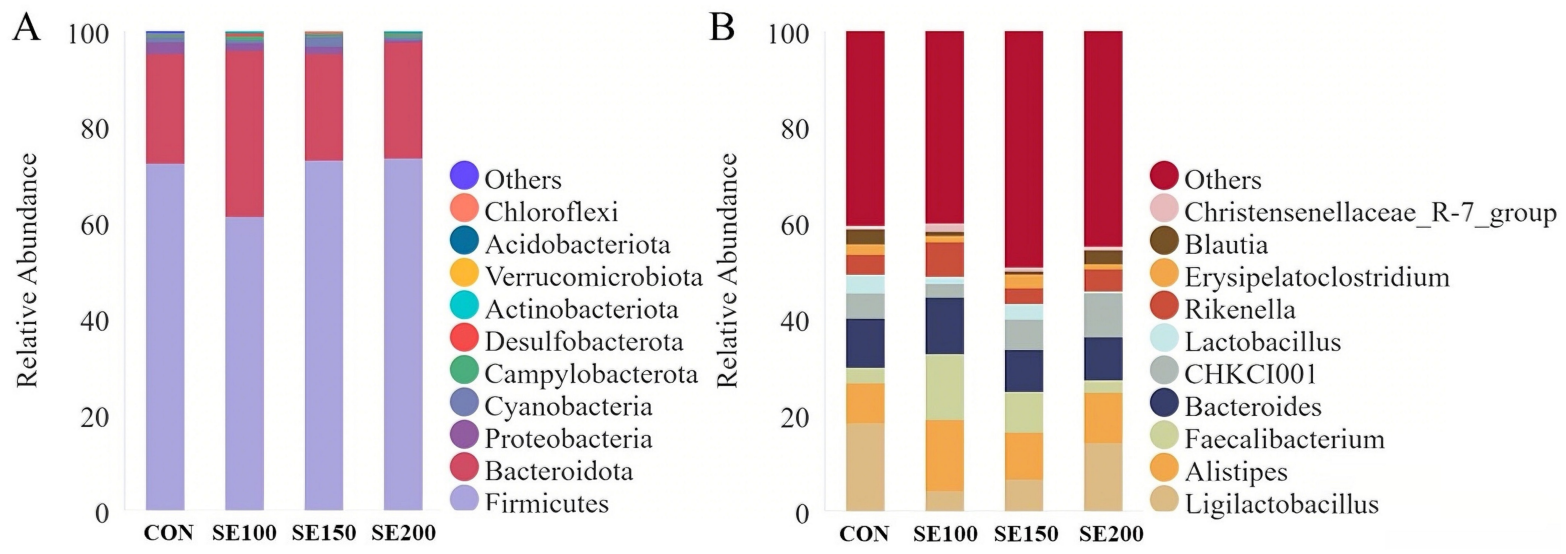
The effects of *Fructus sophorae* extracts on cecal microbiota abundance in broiler chickens. **(A)** The abundance of the TOP10 species at the phylum level. **(B)** The abundance of the TOP10 species at the genus level.

In Table 6, the unit for antioxidant activity in livers was incorrectly labeled. We would like to emphasize that all numerical values and data presented in the table are accurate and based on the original experimental records; only the unit designation was mistakenly entered during the table preparation stage.

The corrected Table 6 appears below:

**Table 6:** The effect of *Fructus sophorae* extract on antioxidant activity of serum and liver in broiler chickens.

Items	Control	SE100	SE150	SE200	SEM	*p*-value		
						Treatment	Linear	Quadratic
Serum								
MDA, nmol/mL	4.61^a^	4.20^b^	3.94^c^	3.96^c^	0.074	<0.001	<0.001	<0.001
SOD, U/mL	125.58^b^	129.97^b^	138.34^a^	138.54^a^	1.584	<0.001	<0.001	<0.001
GSH-Px, U/mL	879.90^b^	930.79^a^	937.33^a^	961.07^a^	8.824	<0.001	<0.001	<0.001
Liver								
MDA, nmol/mg prot	2.73^a^	2.56^b^	2.55^b^	2.58^b^	0.024	0.013	0.033	0.005
SOD, U/mg prot	43.68^b^	46.99^a^	48.52^a^	47.32^a^	0.513	<0.001	0.003	<0.001
GSH-Px, U/mg prot	28.42	28.53	28.58	28.72	0.093	0.751	0.262	0.545

Control, SE100, SE150, and SE200 represent the basal diet supplemented with 0, 100, 150, and 200 mg/kg Fructus sophorae extracts, respectively. SE, Fructus sophorae extracts; MDA, malondialdehyde; SOD, superoxide dismutase; GSH-Px, Glutathione peroxidase. SEM, total standard error of the means. ^*a, b, c*^Mean differ significantly (p < 0.05).

The original version of this article has been updated.

